# Immune dysregulation in preeclampsia: integrative analysis of peripheral transcriptomes and placental single-cell land-scapes

**DOI:** 10.3389/fimmu.2025.1638603

**Published:** 2025-12-01

**Authors:** Shu Zheng, Peng Xu, Huimin Shen, Chang Shu

**Affiliations:** Department of Obstetrics, Obstetrics and Gynaecology Center, The First Hospital of Jilin University, Jilin University, Changchun, China

**Keywords:** preeclampsia, biomarkers, immune dysregulation, single-cell RNA sequencing, transcriptome profiling, placenta, peripheral blood

## Abstract

Preeclampsia (PE) is a pregnancy-specific disorder marked by systemic immune imbalance and placental dysfunction, yet the link between peripheral molecular changes and tissue-level immune alterations remains incompletely understood. In this study, we integrated bulk transcriptomic analysis of peripheral blood from pregnant women at high risk for PE with single-cell RNA sequencing (scRNA-seq) of placental tissues to identify key immune-associated genes and explore their functional relevance. Transcriptome-wide differential expression, immune cell deconvolution, co-expression network analysis, and machine learning–based feature selection led to the identification of five candidate genes. Among them, *TCL1A*, *CLEC2B*, and *LGALS9* exhibited robust expression in both datasets and were subjected to transcriptional and post-transcriptional regulatory network analysis. Single-cell profiling revealed that these genes were distinctly expressed in B cells, natural killer (NK) cells, monocytes, and Hofbauer cells, with functional enrichment in immune activation, cytokine signaling, and immune tolerance pathways. These findings illuminate the molecular mechanisms underlying immune dysregulation in PE and highlight *TCL1A*, *CLEC2B*, and *LGALS9* as promising biomarkers for early detection and mechanistic investigation of the disease.

## 1 Introduction

Preeclampsia (PE) is a pregnancy-specific hypertensive disorder characterized by new-onset hypertension and proteinuria after 20 weeks of gestation, contributing substantially to maternal and perinatal morbidity and mortality worldwide (1, 2). Affecting approximately 2–8% of pregnancies, PE accounts for significant numbers of maternal and fetal deaths annually (3). Although recognized risk factors include nulliparity, multiple gestation, prior history of PE, chronic hypertension, diabetes, renal disease, obesity, advanced maternal age, and autoimmune conditions, the disorder may also occur in women without identifiable risk factors, and its pathogenesis remains incompletely understood (4).

Recent research has increasingly highlighted immune dysregulation as a central feature in the pathogenesis of PE (5). In particular, aberrant immune activation and maternal–fetal immune interactions appear to play a pivotal role in the disorder’s development (6). While the placenta has long been recognized as the central organ involved in PE, the immune mechanisms that drive placental dysfunction are only beginning to be understood. Extravillous trophoblasts fail to adequately remodel uterine spiral arteries, leading to placental hypoperfusion and ischemia, which in turn triggers the release of anti-angiogenic factors, such as soluble Flt-1, and pro-inflammatory cytokines into the maternal circulation (7, 8). These circulating mediators induce systemic endothelial dysfunction, vasoconstriction, and a prothrombotic state, resulting in the clinical manifestations of PE, including hypertension, proteinuria, and multi-organ dysfunction (9).

The widely accepted “two-stage” model posits that placental dysfunction precedes and precipitates the maternal syndrome, the molecular and cellular mechanisms linking these stages are not fully elucidated (10). Despite extensive research, the precise molecular and immune drivers of PE remain elusive. The disease is multifactorial, influenced by genetic, immunological, and environmental factor (11). Notably, immune dysregulation, including altered T-cell responses, macrophage polarization, and cytokine imbalance, contributes significantly to the disease’s pathophysiology (12). However, no definitive biomarkers or targeted therapies are available, and the delivery of the placenta remains the only curative intervention.

Transcriptomic studies of PE have produced heterogeneous results, with limited overlap in differentially expressed genes across cohorts—likely reflecting disease heterogeneity, variable study designs, and population differences (13). This underscores the need for integrative and high-resolution molecular analyses to better delineate the mechanisms underpinning PE.

Recent advances in high-throughput technologies have provided new opportunities to interrogate the molecular landscape of PE (14). Bulk RNA sequencing (RNA-seq) of placental tissue has identified dysregulation of genes involved in angiogenesis, inflammation, and metabolism, but cannot resolve cell type-specific changes (15, 16). Single-cell RNA sequencing (scRNA-seq) overcomes this limitation by enabling transcriptomic profiling at single-cell resolution, thus revealing the cellular heterogeneity of the placenta and highlighting pathogenic alterations in specific trophoblast and immune cell subsets in PE (15, 17, 18).

Given that PE involves both placental and systemic (maternal) alterations, integrative approaches that combine molecular data from placental tissue and maternal blood offer comprehensive insight into disease pathogenesis (19, 20). In this study, we employed a multi-level transcriptomic strategy by performing scRNA-seq on placental samples and bulk RNA sequencing on peripheral blood from women with PE and gestational age-matched controls. By integrating these datasets, we aimed to identify key genes and pathways dysregulated in PE, explore their cellular origins, and elucidate potential links between placental dysfunction and systemic immune responses.

Our findings reveal novel candidate genes and immune-related pathways potentially involved in the immunopathogenesis of PE. This integrative analysis provides new insights into the molecular mechanisms underlying PE and highlights immune dysregulation as a crucial factor in its pathogenesis. These results could facilitate the development of improved biomarkers and therapeutic strategies for this serious pregnancy complication.

## 2 Materials and methods

### 2.1 Sample collection

This study was approved by the Institutional Ethics Committee of the First Hospital of Jilin University (approval number: 2024-HS-145) and conducted in accordance with the Declaration of Helsinki, with written informed consent obtained from all participants. For peripheral blood transcriptomic analysis, samples were collected during the second trimester from 10 pregnant women at increased risk for preeclampsia—defined by one or more established risk factors (including newly elevated blood pressure [≥140/90 mmHg] at the first prenatal visit, history of preeclampsia, body mass index (BMI) ≥35 kg/m², positive antiphospholipid antibodies, chronic hypertension, or a first-degree family history of preeclampsia)—as well as 10 healthy, gestational age-matched controls during routine antenatal outpatient screening. For scRNA-seq, placental tissues were obtained in the third trimester from three early-onset PE patients and two age-matched controls undergoing elective cesarean section, with samples immediately processed to ensure tissue integrity for downstream analysis.

### 2.2 Differential expression analysis

Differential gene expression was assessed using the Limma package (v3.58.1). Raw count data were first transformed by the voom method to stabilize the mean–variance relationship (21). Linear models were then fitted to each gene expression profile, and empirical Bayes moderation was applied to improve variance es-timates. Genes exhibiting a two-sided p-value < 0.05 and an absolute log_2_ fold change (|log_2_FC|) > 1 were deemed significantly differentially expressed. Volcano plots were gener-ated with ggplot2 (v3.5.1) to visualize the overall distribution of fold changes and significance levels, while heat maps of the top 50 up- and down-regulated genes were produced using pheatmap (v1.0.12), with expression values scaled by row. All analyses adhered to the package defaults unless otherwise specified.

### 2.3 Immune infiltration

The relative abundance of infiltrating immune cell subsets was estimated using CIBERSORT (v1.03). Normalized bulk RNA-seq expression matrices were deconvoluted against the LM22 signature matrix via support vector regression. Deconvolution was run with 1,000 permutations, and only samples yielding a CIBERSORT p-value < 0.05 were considered reliable and retained for downstream analysis. Visualization of immune‐infiltration profiles (stacked bar charts and heat maps) was performed using the R packages ggplot2 and pheatmap under default settings.

### 2.4 WGCNA analysis

Weighted gene co-expression networks were constructed using the WGCNA (v1.72.5) (22). To reduce noise, the top 10,000 genes ranked by variance across all samples were retained. A soft-thresholding power of 12 was selected by analyzing scale-free topology criteria (R² > 0.85). The resulting adjacency matrix was transformed into a topological overlap matrix (TOM) to quantify network connectivity. Hierarchical clustering of the TOM was performed using the average linkage method, and modules were detected via dynamic tree cut (minimum module size = 30, merge cut height = 0.25). Each module was assigned a unique color label. Module eigengenes (MEs) were correlated with external traits, including estimated immune cell pro-portions from CIBERSORT and clinical phenotypes, using Pearson correlation. Modules showing significant associations (p < 0.05) were prioritized for further analysis. Within these modules, hub genes were defined as those with high intramodular connectivity (module membership > 0.8) and strong trait correlation (gene significance > 0.2). Identified hub genes were carried forward for downstream functional enrichment and network visualization (23).

### 2.5 Integrated functional and network analysis

The list of intersecting genes was uploaded to the Metascape platform with “Homo sapiens” selected as the species (24). Gene Ontology (GO) pathway enrichment analysis was performed, with statistical significance defined as a minimum overlap ≥3 and p ≤ 0.01. The protein–protein interaction network was constructed using the STRING database (confidence threshold ≥0.7). Significance was evaluated by hypergeometric testing, and results were corrected for multiple testing using the false discovery rate (FDR) method. Only pathways with FDR < 0.1 were retained, and genes without functional annotation were excluded. Core nodes within the network were identified based on a degree centrality ≥5, thereby determining key regulatory nodes.

### 2.6 Feature selection process of Lasso regression and SVM algorithm

To pinpoint robust diagnostic biomarkers, we employed two complementary ma-chine-learning approaches: LASSO logistic regression and support vector machine recursive feature elimination (SVM-RFE) (25). LASSO logistic regression was performed using the glmnet (v4.1.8). The expression matrix of candidate genes served as input, with disease status as the outcome. We applied the cv.glmnet() function with ten-fold cross-validation to determine the optimal penalty parameter (λ) that minimized the mean cross-validated error. Genes with nonzero coefficients at this λ were selected as LASSO‐derived features. SVM-RFE was conducted using the e1071 R package (v1.7.14). A linear‐kernel SVM was trained on the same ex-pression data. Recursive feature elimination proceeded by iteratively removing the least con-tributory genes—ranked by absolute weight magnitude—and evaluating model accuracy via ten-fold cross-validation at each step. The gene subset achieving the highest classification accuracy was retained. The intersection of LASSO‐ and SVM-RFE‐selected genes was con-sidered the final panel of candidate diagnostic markers for downstream validation.

### 2.7 GSEA analysis

Patients were stratified into high- and low-expression groups for each core gene using the median expression as the cutoff. Gene set enrichment analysis (GSEA) was performed using the clusterProfiler package. A pre-ranked list of all genes—ordered by log_2_ fold change be-tween high- and low-expression groups—was submitted to the GSEA() function. Gene sets were drawn from the MSigDB Hallmark and KEGG collections. Analysis parameters included 1,000 phenotype-based permutations (nPerm = 1000, permType = “phenotype”). Enriched pathways with nominal p-value < 0.05 and Benjamini–Hochberg–adjusted q-value < 0.05 were considered significant. Enrichment plots were generated using the gseaplot2() function.

### 2.8 Quality control and filtering criteria

Raw UMI count matrices were imported into Seurat (v4.3.0) for quality control, during which four key metrics—library complexity (total UMI counts and number of detected genes), mito-chondrial transcript fraction (percentage of reads mapping to MT-encoded genes), and ribo-somal transcript fraction (percentage of reads mapping to RPS/RPL gene families)—were computed for each cell (26). To exclude low-quality or apoptotic cells, we removed any cell with fewer than 200 or more than 6,500 detected genes, fewer than 500 or more than 50,000 UMIs, mitochondrial content exceeding 10%, or ribosomal content exceeding 30%. These thresholds were selected based on inspection of metric distributions (via violin and scatter plots) to dis-card outliers beyond 1.5× the interquartile range. Following filtering, the retained cells dis-played high library complexity and minimal stress indicators, providing a robust dataset for subsequent normalization and downstream analyses.

### 2.9 Data standardization and cell annotation

After quality control, expression matrices were normalized and scaled in Seurat using the “LogNormalize” method, whereby each cell’s counts were divided by the total counts, multi-plied by a scale factor of 10,000, and log‐transformed. Cell‐cycle heterogeneity was assessed with CellCycleScoring, and the top 2,000 highly variable genes were identified using Find-VariableFeatures. To minimize technical and biological confounders, we applied ScaleData to regress out mitochondrial gene percentage, ribosomal gene percentage, and cell‐cycle scores. Linear dimensionality reduction was then performed via principal component analysis (PCA), and Harmony integration was applied to the principal components (PCs) to correct batch effects across samples. Nonlinear dimensionality reduction for visualization was achieved with Uniform Manifold Approximation and Projection (UMAP) on the Harmo-ny-corrected PCs, and graph‐based clustering (FindNeighbors/FindClusters) delineated dis-crete cell populations. Finally, clusters were annotated by cross‐referencing cluster marker genes with published literature and entries in the CellMarker database, enabling precise iden-tification of trophoblast, immune, and stromal subsets relevant to preeclampsia pathogenesis.

### 2.10 Statistical analysis

Statistical analyses were conducted in R (v4.2.1). Unless otherwise noted, all tests were two‐sided, and p-values below 0.05 were considered statistically significant.

## 3 Results

### 3.1 Peripheral blood transcriptomic and immune profiling in pregnant women at risk for PE

To investigate early molecular and immunological alterations associated with PE, we first per-formed transcriptomic profiling of peripheral blood samples from 20 pregnant women, com-prising 10 individuals at increased risk for PE and 10 healthy, gestational age-matched controls. The PE-risk group was defined by the presence of one or more established risk factors, in-cluding newly elevated blood pressure (≥140/90 mmHg) at the initial prenatal visit, prior history of PE, maternal age ≥40 years, body mass index (BMI) ≥35 kg/m², positive antiphos-pholipid antibodies, chronic hypertension, or a first-degree family history of PE. All samples were obtained during routine antenatal outpatient screening.

**Figure 1:**
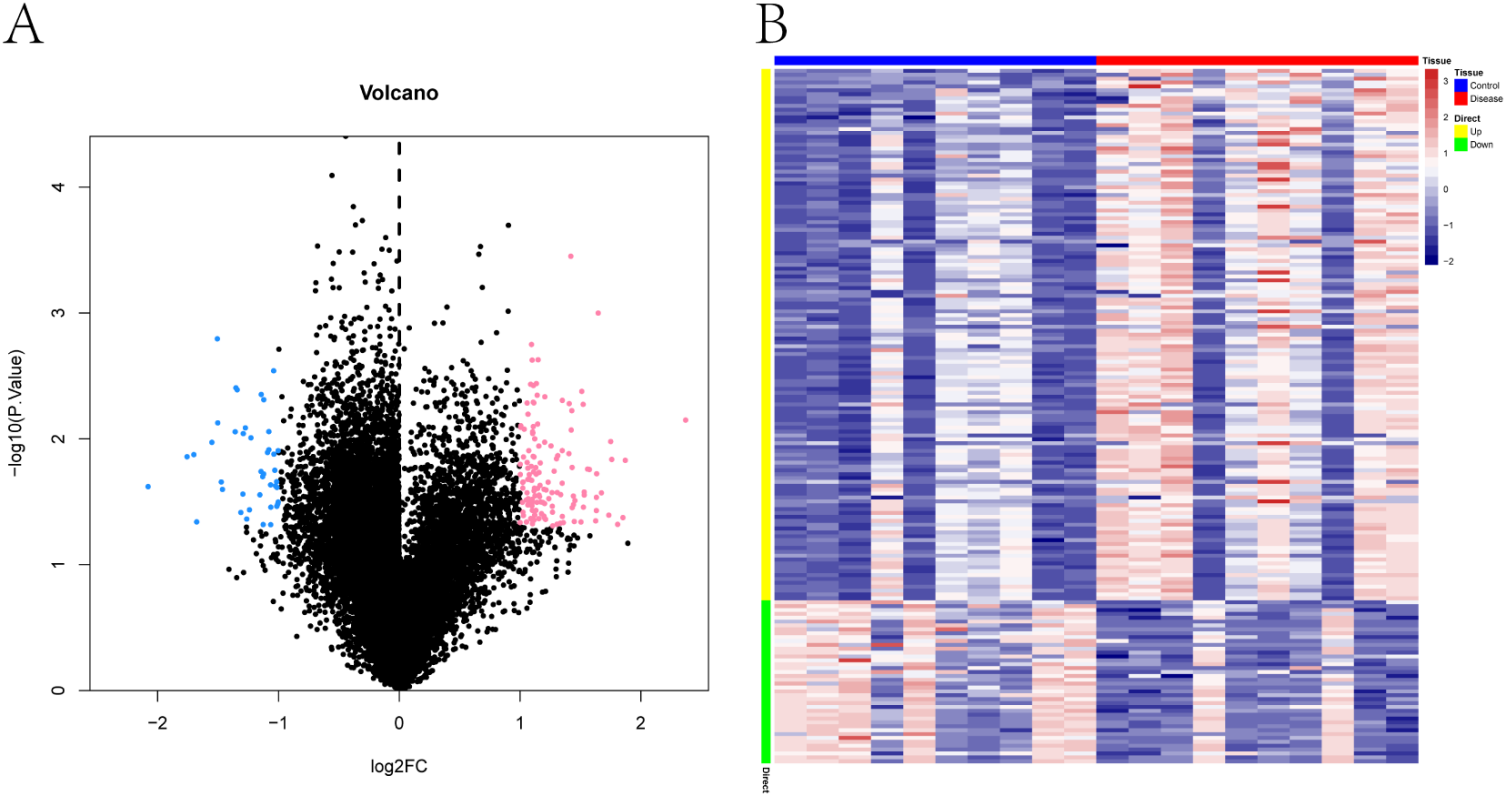
Transcriptomic differences in peripheral blood between pregnant women at risk for PE and healthy controls. **(A)** Volcano plot of differentially expressed genes (DEGs) between PE-risk and control groups. Each dot represents a gene. Genes with p < 0.05 and |log_2_FC| > 1 are shown in pink (upregulated) and blue (downregulated); non-significant genes are shown in black. **(B)** Heatmap showing the expression profiles of DEGs across all peripheral blood samples. Rows represent genes, and columns represent individual samples from PE-risk (n = 10) and control (n = 10) pregnancies. Red indicates high expression; blue indicates low expression.

Differential gene expression analysis was conducted using the Limma package. With the thresholds set at p < 0.05 and |log_2_ fold change| > 1, a total of 179 differentially expressed genes (DEGs) were identified between the PE-risk and control groups, including 137 upregu-lated and 42 downregulated genes in the PE-risk cohort. A volcano plot illustrating the dis-tribution of DEGs is presented in **Figure 1A**, where pink and blue indicate upregulated and downregulated genes, respectively. The accompanying heatmap (**Figure 1B**) demonstrates distinct gene expression patterns between the two groups, with red representing higher and blue representing lower expression levels. These results indicate that substantial transcrip-tional alterations are already evident in the peripheral blood of women at risk for PE.

**Figure 2:**
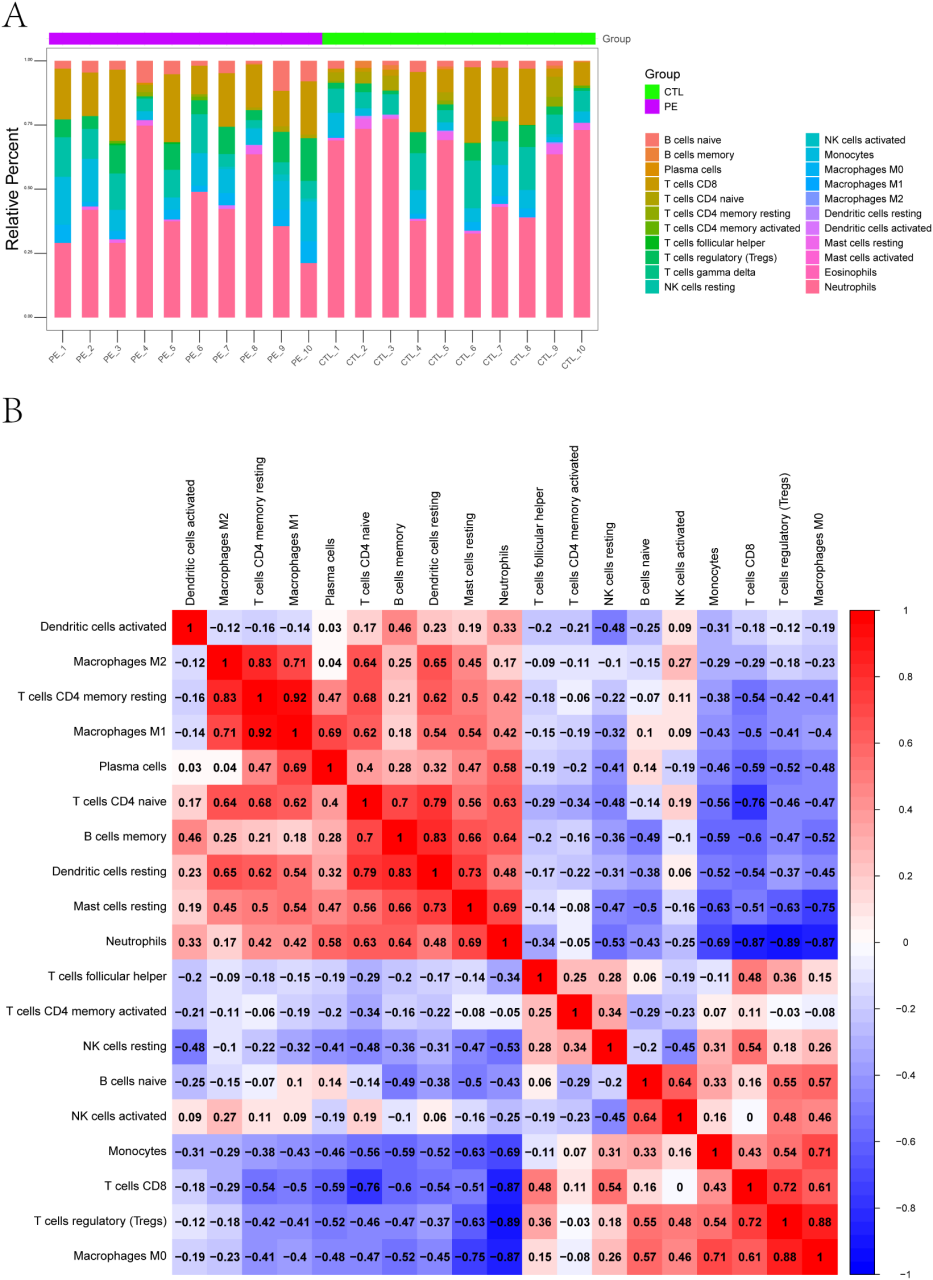
Immune landscape characterization of peripheral blood in pregnant women at risk for PE. **(A)** Relative proportions of 22 immune cell subtypes estimated by CIBERSORT. Stacked bar plots display the distribution of immune cell types in each peripheral blood sample from PE-risk and control groups. **(B)** Correlation heatmap of immune cell subtypes. Spearman cor-relation coefficients were calculated based on the estimated proportions of immune subsets. Positive correlations are shown in red, and negative correlations in blue.

To further explore the immune microenvironment, we employed the CIBERSORT algorithm to estimate the relative abundance of 22 immune cell subtypes from bulk transcriptomic data. The immune cell composition of each sample is shown as a stacked bar chart (**Figure 2A**), revealing notable differences between PE-risk and control pregnancies. In particular, the PE-risk group exhibited increased proportions of monocytes and activated dendritic cells, along with decreased fractions of regulatory T cells (Tregs) and resting NK cells, indicating a shift toward a pro-inflammatory peripheral immune profile.

We additionally assessed the correlations among immune cell subsets using CIBERSORT-derived proportions. The correlation heatmap (**Figure 2B**) revealed a network of interactions among immune populations. Positive correlations were observed among activated NK cells, monocytes, and CD8^+^ T cells, while Tregs were negatively correlated with M1 macrophages and neutrophils. These patterns reflect a potential imbalance between regulatory and effector immune responses, which may underlie early immune activation in pregnancies predisposed to PE.

### 3.2 WGCNA identifies a neutrophil-associated gene module enriched in immune regulatory pathways

**Figure 3:**
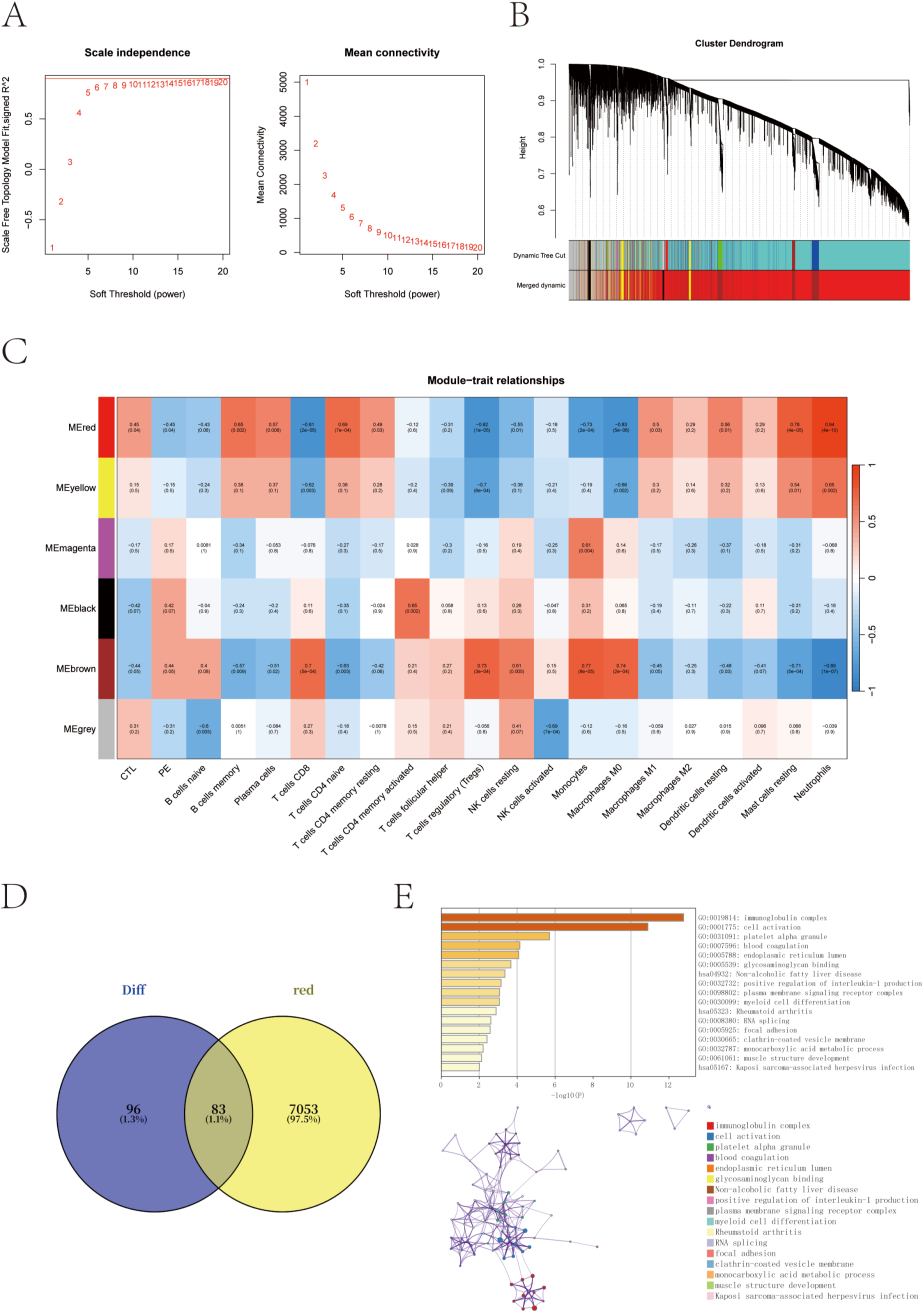
Co-expression network construction and trait correlation analysis via WGCNA. **(A)** Determination of the optimal soft-thresholding power (β = 14) based on scale-free topology fit index and mean connectivity. **(B)** Gene dendrogram obtained by hierarchical clustering of the topological overlap matrix (TOM); colors represent different co-expression modules. **(C)** Heatmap of correlations between module eigengenes and clinical/immune traits. The red module shows the strongest positive correlation with neutrophil scores and PE-risk status. **(D)** Venn diagram showing the overlap between red module genes and previously identified differentially expressed genes (DEGs). **(E)** Functional enrichment of the 83 overlapping genes using the GO and KEGG enrichment analysis through the Metascape database.

To further explore the regulatory networks underlying transcriptional alterations in pregnant women at risk for PE, we performed weighted gene co-expression network analysis (WGCNA) based on scale-free topology criteria (**Figure 3A**). Hierarchical clustering based on the topological overlap matrix (TOM) identified six gene co-expression modules, labeled by color as black (154 genes), brown (1,430), grey (911), magenta (59), red (7,136), and yellow (310) (**Figure 3B**).

Module–trait correlation analysis revealed that the red module was most strongly associated with PE-risk status and with neutrophil infiltration scores (cor = 0.94, p = 4 × 10^-10^), suggesting its potential involvement in immune dysregulation linked to PE susceptibility (**Figure 3C**). To narrow down the candidate gene set, we intersected the red module genes with the previously identified DEGs, yielding a total of 83 overlapping genes (**Figure 3D**).

Functional enrichment analysis of these 83 intersection genes was performed to gain insights into their biological relevance. The analysis revealed significant enrichment in immune-related pathways, particularly in the immunoglobulin complex, along with terms related to cell activation and myeloid cell differentiation. These findings underscore the central role of immune dysregulation in the pathogenesis of preeclampsia (**Figure 3E**).

### 3.3 Identification of core candidate genes by LASSO and SVM algorithms

**Figure 4:**
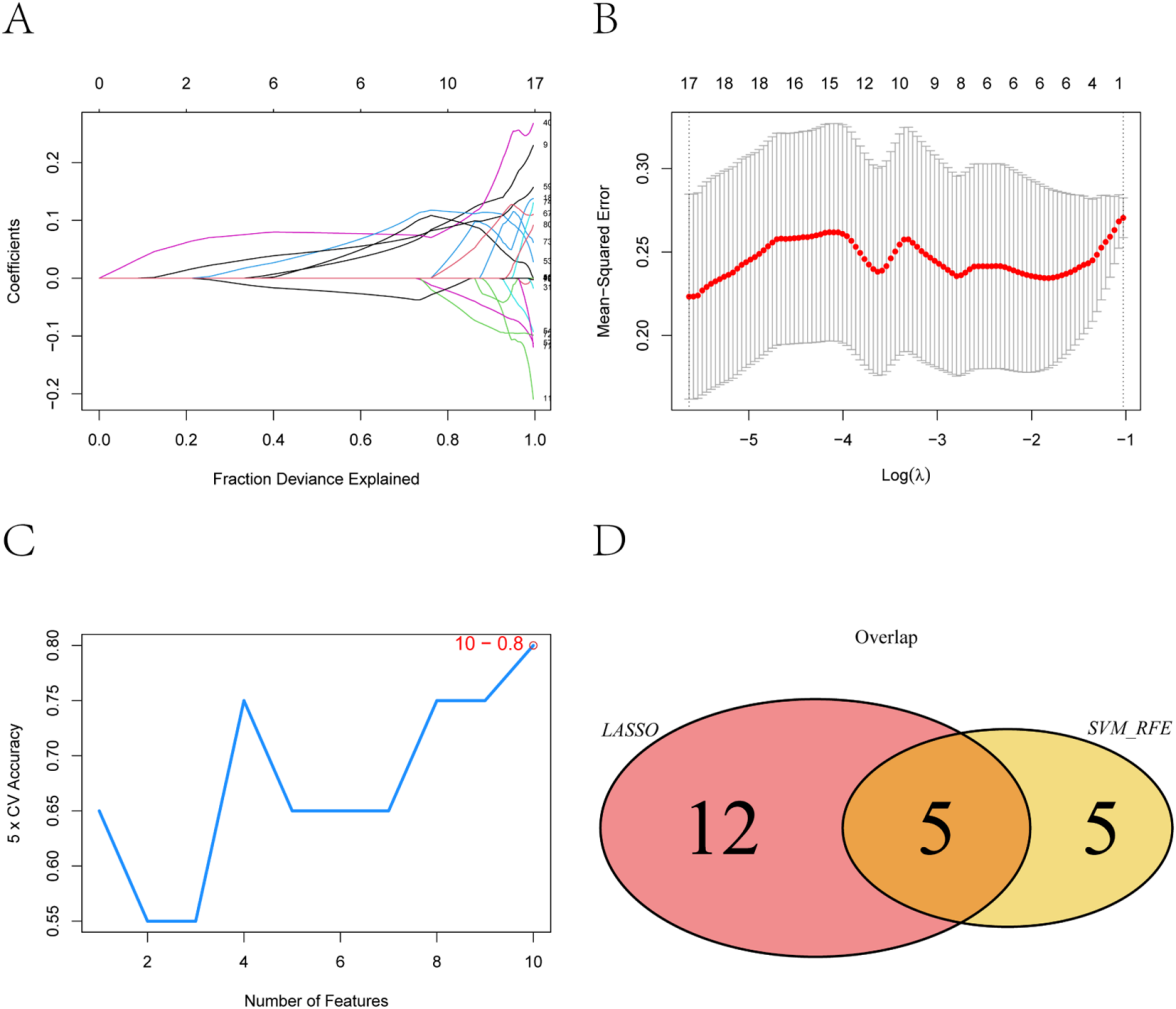
Feature selection of key genes using LASSO and SVM-RFE algorithms. **(A)** Coefficient distribution of the 83 input genes and the selected gene set at the optimal lambda (λ) value. **(B)** Ten-fold cross-validation in the LASSO model for tuning parameter selection and determination of the optimal lambda (λ). **(C)** Top 10 feature genes corresponding to the highest classification accuracy in the SVM-RFE model. **(D)** Intersection of feature genes selected by the LASSO regression and SVM-RFE algo-rithms.

To further narrow down potential biomarkers relevant to PE, we applied two supervised machine learning algorithms—LASSO regression and support vector machine-recursive feature elimination (SVM-RFE)—to the 83 intersecting genes identified from the neutrophil-associated co-expression module and differential expression analysis. These genes represent a subset of immune-related transcripts that are both co-expressed and differentially regulated in pregnant women at risk for PE, suggesting functional relevance to disease development. LASSO regression identified 17 genes with non-zero coefficients as optimal features for distinguishing PE-risk individuals from controls, as determined by the minimum cross-validation error (**Figures 4A, B**). Meanwhile, SVM-RFE analysis ranked the top 10 genes with the highest classification accuracy (**Figure 4C**). By intersecting both sets, five genes—*TCL1A*, *CLEC2B*, *LGALS9*, *IGLV6-57*, and *AL450405.1* —were consistently identified (**Figure 4D**) and defined as robust candidate biomarkers for further analysis.

### 3.4 Immune regulatory associations of the core genes

**Figure 5:**
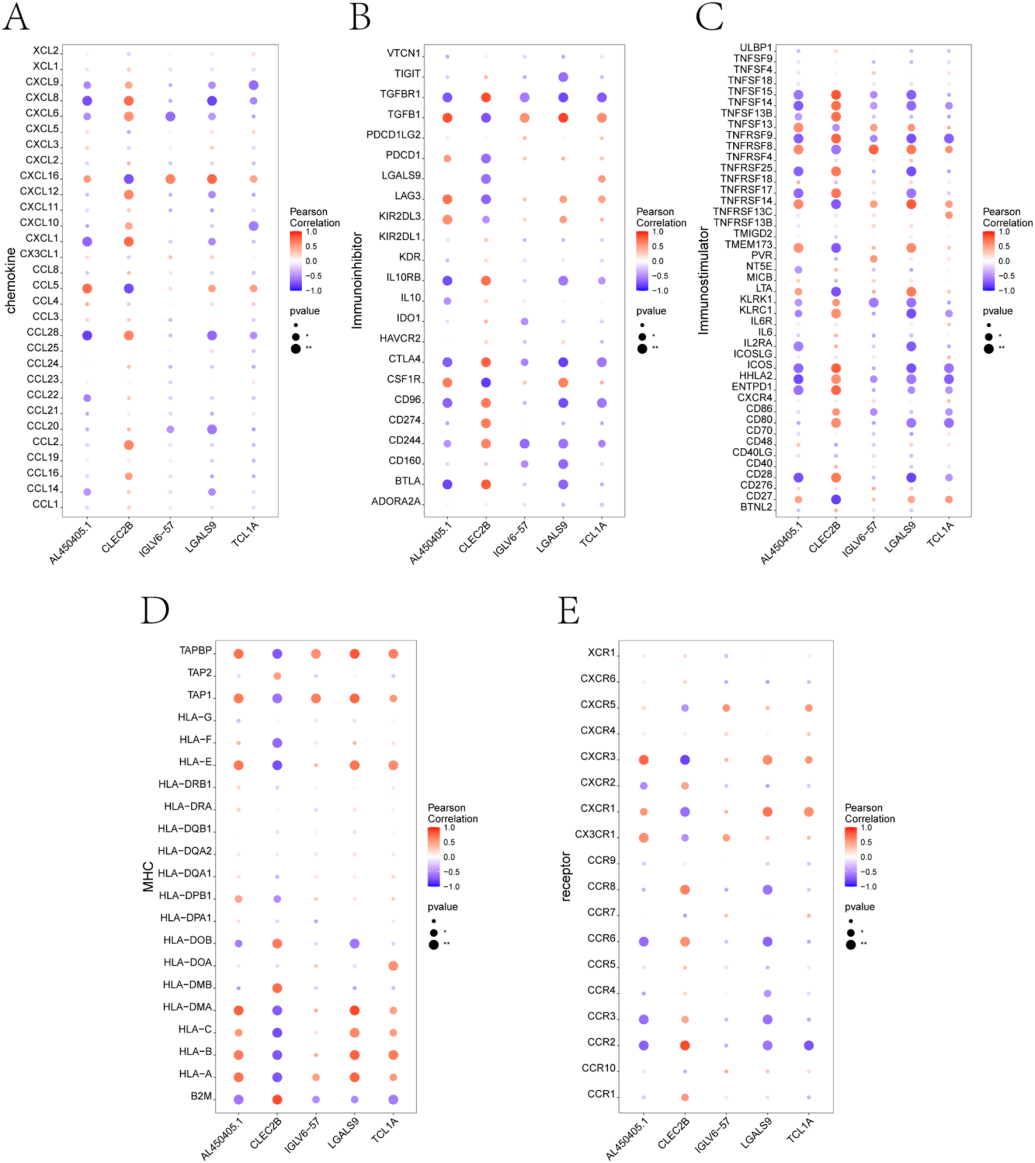
Correlation analysis between key genes and immune-related factors using TISIDB. **(A-E)** Correlation between the five core genes (*TCL1A*, *CLEC2B*, *LGALS9*, *IGLV6-57*, *AL450405.1*) and different categories of immune regulatory factors, including: **(A)** Chemokines, **(B)** Immunoinhibitors, **(C)** Immunostimulators, **(D)** Major histocompatibility complex (MHC) molecules, and **(E)** Immune-related receptors. Each dot represents the correlation between a single gene and an immune molecule. Red indicates positive correlation, and blue indicates negative correlation. Dot size reflects p-value significance.

To explore how the five identified candidate genes might contribute to immune dysregulation in preeclampsia, we evaluated their correlations with immune-related molecules using the TISIDB database. The correlation heatmaps revealed that all five genes—*TCL1A*, *CLEC2B*, *LGALS9*, *IGLV6-57*, and *AL450405.1*—were significantly associated with numerous immune components (**Figures 5A–E**). Several genes showed notable positive or negative correlations with chemokines and immune receptors, suggesting potential involvement in immune cell recruitment and signaling. In addition, correlations with key immunomodulators and MHC molecules indicate their possible roles in shaping antigen presentation and immune tolerance (27). These findings highlight the relevance of the core genes in modulating both innate and adaptive immune responses, reinforcing their potential contribution to the immunological landscape of PE (28).

### 3.5 GSEA-based identification of signaling pathways involving core genes

To further investigate the potential molecular mechanisms through which the five core genes may influence preeclampsia progression, we performed gene set enrichment analysis (GSEA) using KEGG pathway sets. For each gene, we visualized the enrichment of biological pathways based on expression-level–ranked gene profiles.

**Figure 6:**
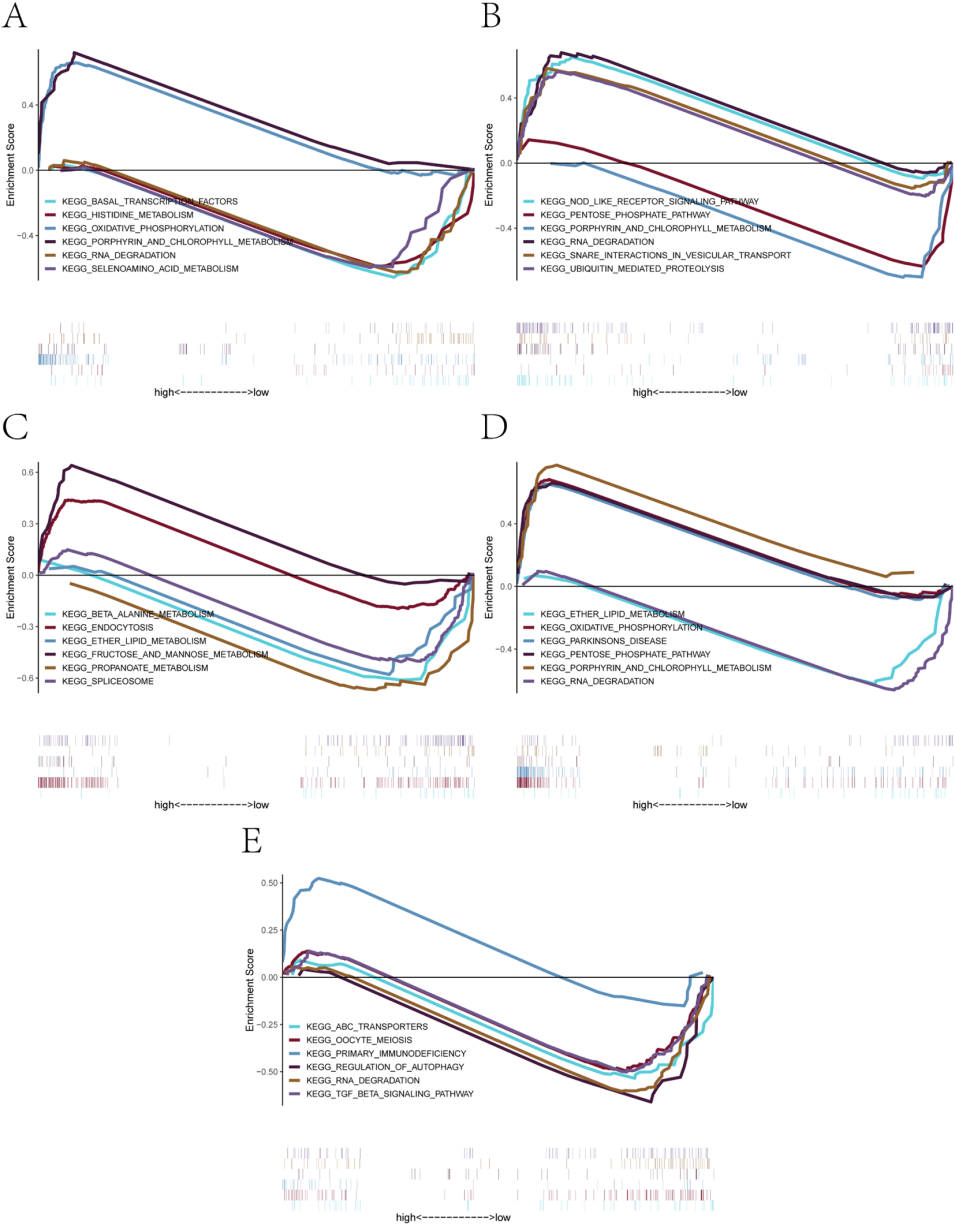
GSEA-based KEGG pathway enrichment analysis of the five core genes. **(A-E)** Significantly enriched KEGG signaling pathways associated with: **(A)***AL450405.1*, **(B)***CLEC2B*, **(C)***IGLV6-57*, **(D)***LGALS9*, **(E)***TCL1A*. Each plot shows positively or negatively enriched pathways between high- and low-expression groups, along with their enrichment scores and contributing genes.

The GSEA results revealed distinct enrichment profiles across genes (**Figures 6A–E**). Specifically, *AL450405.1* was enriched in signaling pathways such as basal_transcription_factors, histidine_metabolism, and oxidative_phosphorylation (**Figure 6A**). CLEC2B was enriched in nod_like_receptor_signaling_pathway, pentose_phosphate_pathway, and rna_degradation (**Figure 6B**). *IGLV6–57* showed enrichment in beta_alanine_metabolism, endocytosis, and ether_lipid_metabolism (**Figure 6C**). *LGALS9* was enriched in oxidative_phosphorylation, parkinsons_disease, and rna_degradation (**Figure 6D**). Finally, *TCL1A* was associated with abc_transporters, oocyte_meiosis, and primary_immunodeficiency (**Figure 6E**). These findings indicate that the five core genes are involved in a wide range of biological pathways, including those related to transcriptional regulation, immune signaling, energy metabolism, and cell cycle control, providing mechanistic insights into their potential roles in the pathogenesis of PE (29, 30).

### 3.6 Cell-type–resolved expression patterns and immune functional mechanisms in the PE placenta

**Figure 7:**
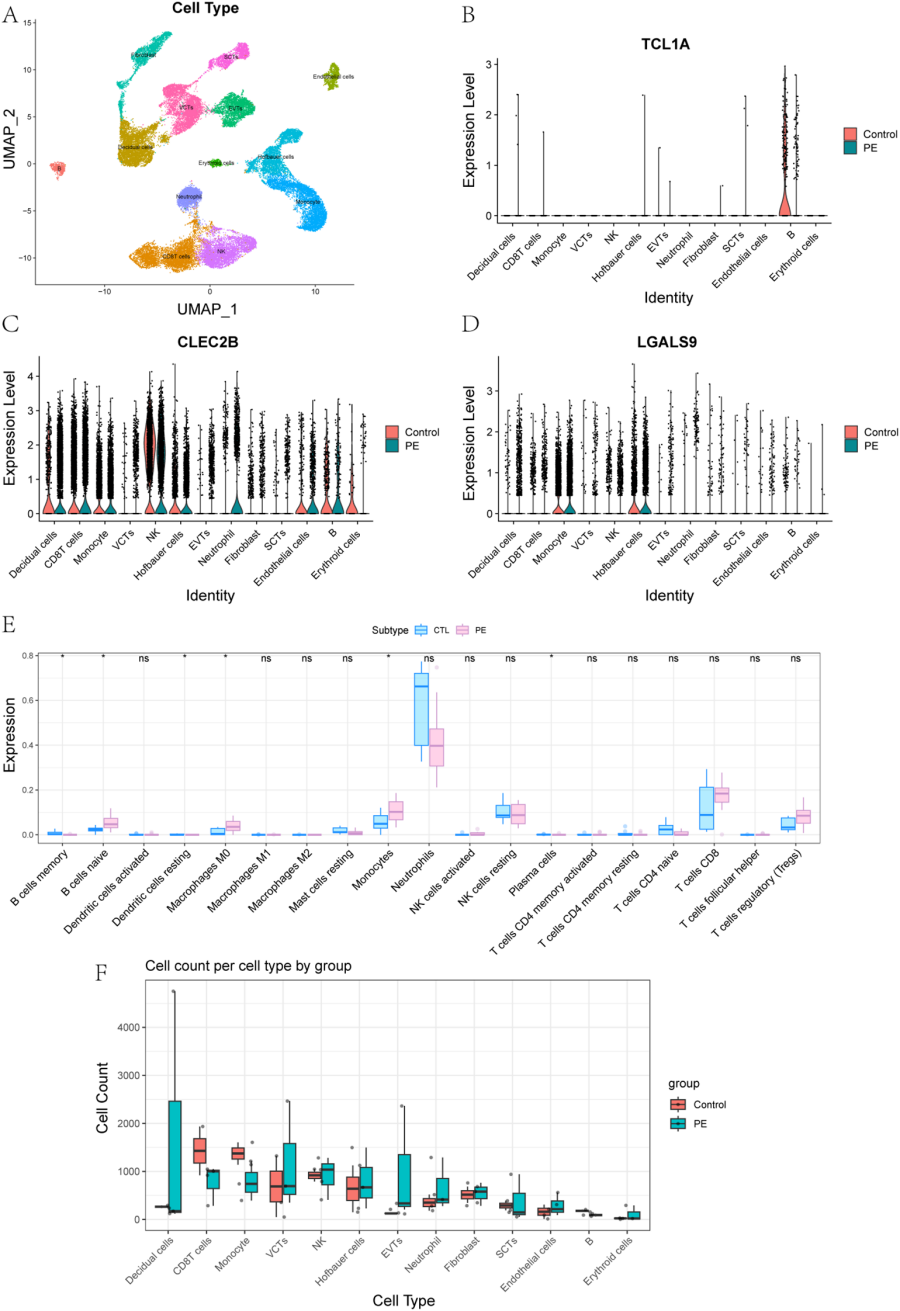
Single-Cell Expression Patterns and Immune Cell Composition in PE and Controls. **(A)** Placental cell types were defined by annotation of clusters based on canonical marker gene expression. **(B-D)** Expression of key genes in 13 placental cell types between control and PE groups: **(B)***TCL1A*, **(C)***CLEC2B*, **(D)***LGALS9*. **(E)** Comparison of immune cell content in peripheral blood transcriptomes between control and PE groups. **(F)** Comparison of the abundance of 13 placental cell types between control and PE groups.

To integrate bulk transcriptomic findings with cell-specific resolution, we conducted single-cell RNA sequencing on placental tissues from three patients with early onset PE and two healthy controls. After rigorous quality control, a total of 41,395 high-quality cells were retained for analysis. UMAP dimensionality reduction revealed 13 major placental cell types, including multiple trophoblast and immune subsets (**Supplementary Figure S1**; **Figure 7A**). Marker gene analysis confirmed accurate cell-type annotation, and analysis of cell proportions revealed notable shifts in immune cell populations between PE and control placentas (**Supplementary Figure S2**).

To further explore the roles of immune-related genes identified in the bulk transcriptomic analysis, we focused on three core genes—*TCL1A*, *CLEC2B*, and *LGALS9*—that displayed distinct cell-type–specific expression patterns in single-cell data. UMAP and violin plots revealed highly distinct and spatially localized expression patterns among these genes (**Supplementary Figure S3**, **Figures 7B-D**). *TCL1A* expression was highly enriched in placental B cells, with significantly higher expression in controls compared to PE. It is well recognized that abnormal B cell activation may lead to the production of autoantibodies or proinflammatory factors, both of which have been implicated in the development of PE. Given its strong expression in B cells, TCL1A may serve as a biomarker for adaptive immune disturbances in PE. *CLEC2B* was predominantly expressed in natural killer (NK) cells. NK cells play a critical role in the regulation of trophoblast invasion and uterine vascular remodeling through cytotoxicity and cytokine production, and aberrant *CLEC2B* expression could reflect altered NK cell function, contributing to defective vascular adaptation and immune imbalance at the maternal–fetal interface (31). This makes CLEC2B a potential marker for immune dysfunction and defective placental vascularization. *LGALS9* was most abundant in Hofbauer cells and monocytes, indicating a role in macrophage-mediated immune modulation within the placenta. *LGALS9*, a galectin family protein, is known to regulate immune checkpoint pathways such as TIM-3, and its dysregulation could disrupt maternal–fetal tolerance, leading to heightened inflammatory responses characteristic of PE (32). These findings suggest that *TCL1A*, *CLEC2B*, and *LGALS9* are not only key players in immune dysregulation in PE but also have potential as early biomarkers for the disease, with implications for early detection and mechanism research.

Additionally, Boxplot analyses of immune cell content across 13 placental cell types revealed significant differences between PE and control groups (**Figures 7E, F**). These analyses indicated that the immune cell composition in the PE placenta was altered compared to the control placenta, supporting the hypothesis that immune dysregulation is a critical factor in the pathogenesis of PE.

## 4 Discussion

PE is a pregnancy-specific disorder characterized by systemic vascular dysfunction and immune dysregulation, yet its molecular pathogenesis remains incompletely understood (33). In this study, we adopted a multi-omics strategy that integrates peripheral blood transcriptome profiling, transcriptional regulatory inference, and scRNA-seq of placental tissue to identify and functionally characterize key genes involved in PE. This approach enabled the discovery of molecular targets with diagnostic potential and mechanistic relevance, offering a high-resolution view of PE-associated immune alterations across both systemic and tissue levels.

Peripheral blood transcriptomic analysis revealed widespread immune and metabolic dysregulation in pregnant women at high risk for PE. Differentially expressed genes were significantly enriched in neutrophil-associated pathways and inflammatory signaling cascades, aligning with the established contribution of innate immune activation to PE pathophysiology. Previous studies have similarly found that neutrophil activation is a key player in immune responses in PE and hypertensive disorders of pregnancy, indicating a potential link to the vascular dysfunction that characterizes the disease (34). Network-based analysis using WGCNA further identified neutrophil-related gene modules tightly correlated with disease status. Subsequent machine learning–based feature selection (LASSO and SVM algorithms) narrowed this list to five candidate genes with strong discriminatory potential. Among these, *TCL1A*, *CLEC2B*, and *LGALS9* were consistently highlighted across multiple analytic layers, underscoring their robust association with PE. Moreover, Previous mechanistic reports on *TCL1A*, *CLEC2B*, and *LGALS9* support their roles in immune regulation, particularly in inflammatory responses, highlighting their potential as disease-relevant biomarkers and providing mechanistic entry points for future functional studies (35, 36).

Mapping the expression of candidate genes to specific placental cell types enabled us to explore how systemic molecular changes may relate to local pathophysiological processes in PE. The cell-type–specific distribution of *TCL1A*, *CLEC2B*, and *LGALS9* suggests that distinct immune and stromal populations within the placenta contribute differentially to disease development. *TCL1A*, expressed exclusively in B cells, highlights the potential involvement of adaptive immune perturbation in placental dysfunction. *CLEC2B*, enriched in natural killer (NK) cells and monocytes, points to excessive cytotoxic signaling and innate immune activation—factors known to impair trophoblast invasion and spiral artery remodeling (37). *LGALS9*, expressed in Hofbauer cells and monocytes, may participate in modulating maternal–fetal immune tolerance via immune checkpoint pathways. These findings provide a mechanistic link between immune dysregulation observed in peripheral blood and alterations in the immune microenvironment of the placenta.

The convergence of systemic transcriptional dysregulation with cell-type–resolved functional engagement underscores the translational potential of the identified genes. *TCL1A*, *CLEC2B*, and *LGALS9* not only exhibited measurable expression changes in peripheral blood but also mapped to immunologically relevant placental cell types, linking maternal signals with tissue-level dysfunction (35, 36). Their association with immune activation, disruption of immune tolerance, and angiogenic pathways strengthens their candidacy as early biomarkers for PE risk assessment. Importantly, the presence of these signals in maternal blood prior to clinical diagnosis suggests that they may serve as accessible indicators of subclinical placental stress, offering opportunities for timely risk stratification and intervention. These findings are consistent with existing literature on early biomarkers for PE, further supporting the utility of immune-related molecules as potential predictive markers in hypertensive disorders of pregnancy.

However, it is important to acknowledge the limitations of this study arising from the biological and clinical heterogeneity of the high-risk cohort. While this diversity enhances the clinical applicability of the study, it also introduces variability that could influence the molecular characteristics identified. Different risk factors may activate distinct pathogenic mechanisms, which may extend beyond immune dysregulation alone. This heterogeneity could impact the ability to detect consistent molecular markers across the entire cohort, particularly in cases where certain risk factors—such as vascular dysfunction or autoimmune activation—might dominate the pathogenesis of PE.

In addition, this study primarily included early-onset preeclampsia cases, a clinically and biologically distinct subtype characterized by more pronounced placental insufficiency and unique immune activation patterns (38). Focusing on early-onset cases provides mechanistic insight into this severe phenotype, and the findings help elucidate immune alterations that may precede or accompany placental dysfunction. While we have acknowledged this heterogeneity as a limitation, future studies that include larger cohorts and explore the potential impact of specific risk factors or subgroups will help refine our understanding of the molecular features of PE.

In summary, this study delineates a multi-layered framework for understanding PE through the integration of bulk transcriptomic profiling, regulatory network inference, and single-cell validation. By identifying three robust candidate genes—*TCL1A*, *CLEC2B*, and *LGALS9*—with consistent associations across systemic and placental compartments, we provide mechanistic insight into the immunological perturbations that drive PE and propose biologically plausible molecular targets for early detection. These findings illustrate the power of combining multi-omics data to uncover clinically relevant markers in complex pregnancy-related disorders.

## Data Availability

The datasets used and analyzed during this study are available from the corresponding author upon reasonable request. The raw sequencing data and processed gene expression data have been deposited in the Gene Expression Omnibus (GEO) database: placental samples from PE patients and controls are available under accession numbers GSE282038, GSE267340 and GSE298119. The peripheral blood transcriptomic data are available under accession number GSE296973.
