# Supplementary figures and images for: Immune dysregulation in preeclampsia: integrative analysis of peripheral transcriptomes and placental single-cell land-scapes
Source: Front Immunol. 2025 Dec 1;16:1638603. doi: 10.3389/fimmu.2025.1638603 (PMC12702750; doi:10.3389/fimmu.2025.1638603)

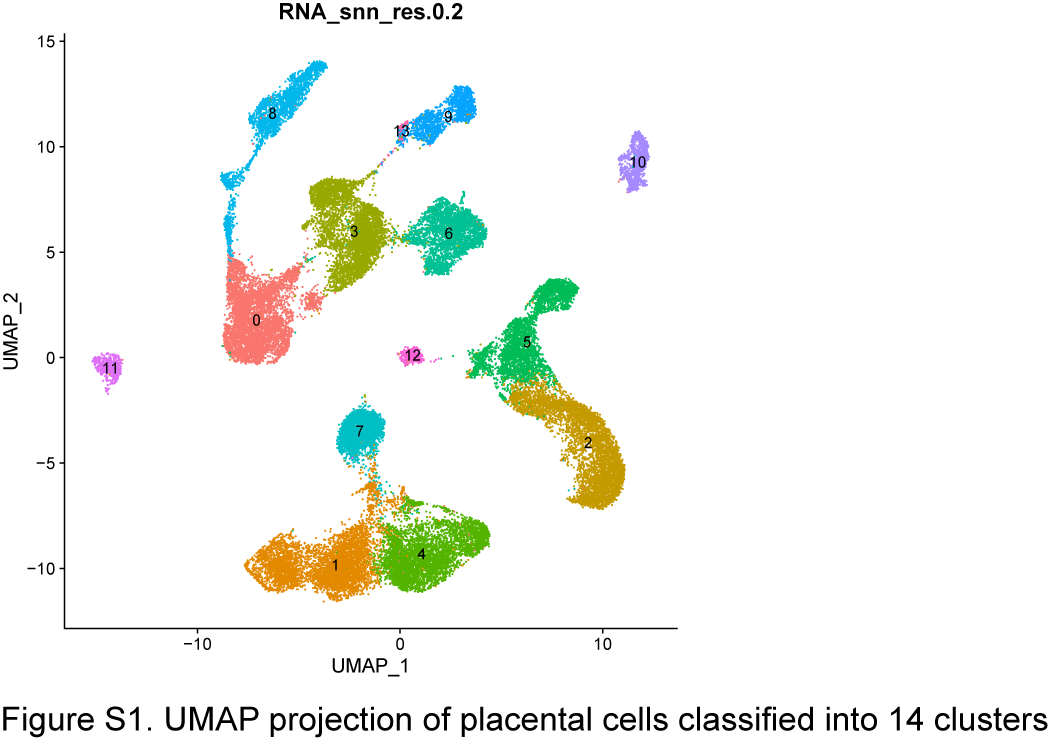

Supplement: Supplementary file 1 [file Image1.tif]

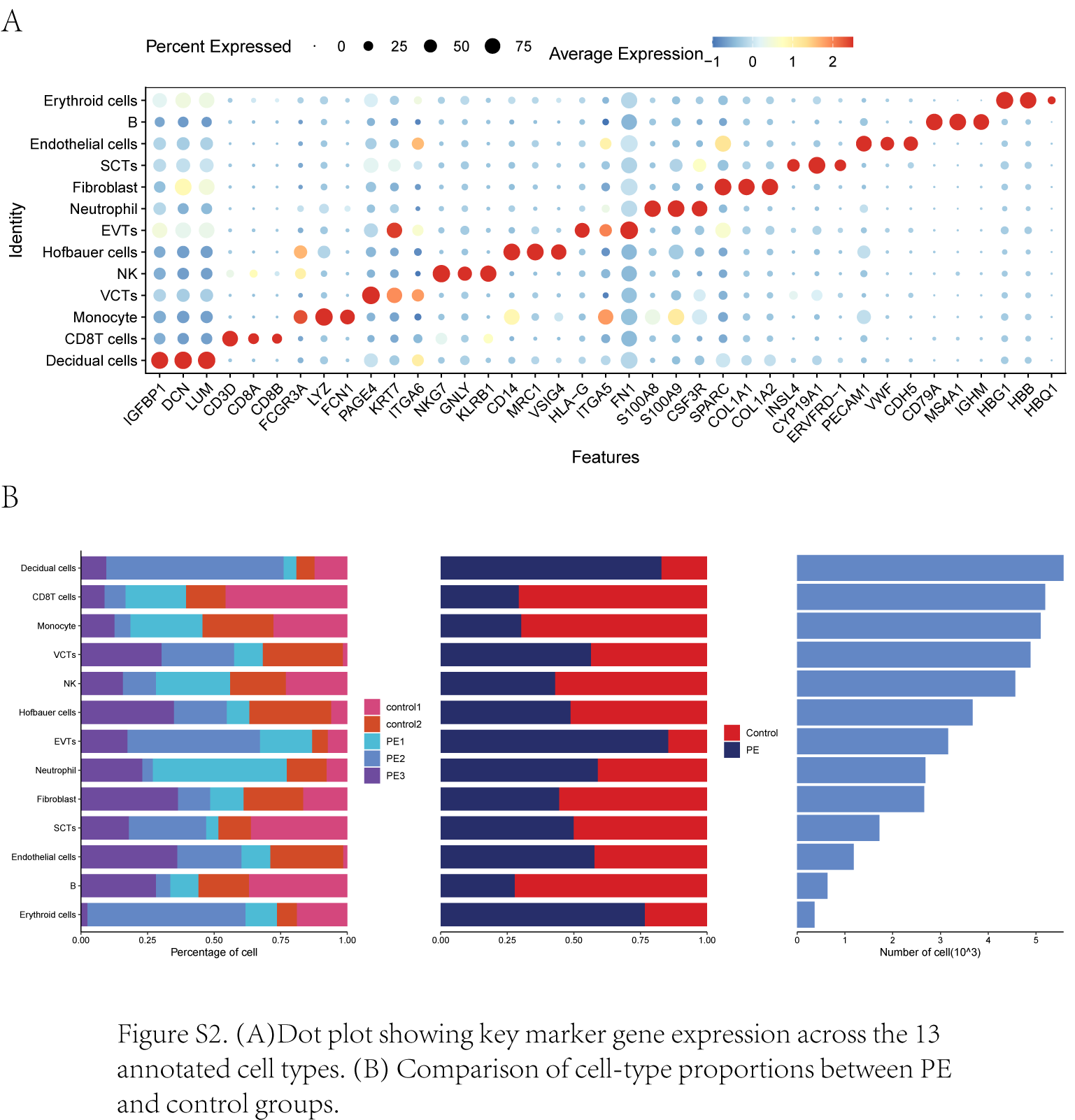

Supplement: Supplementary file 2 [file Image2.tif]

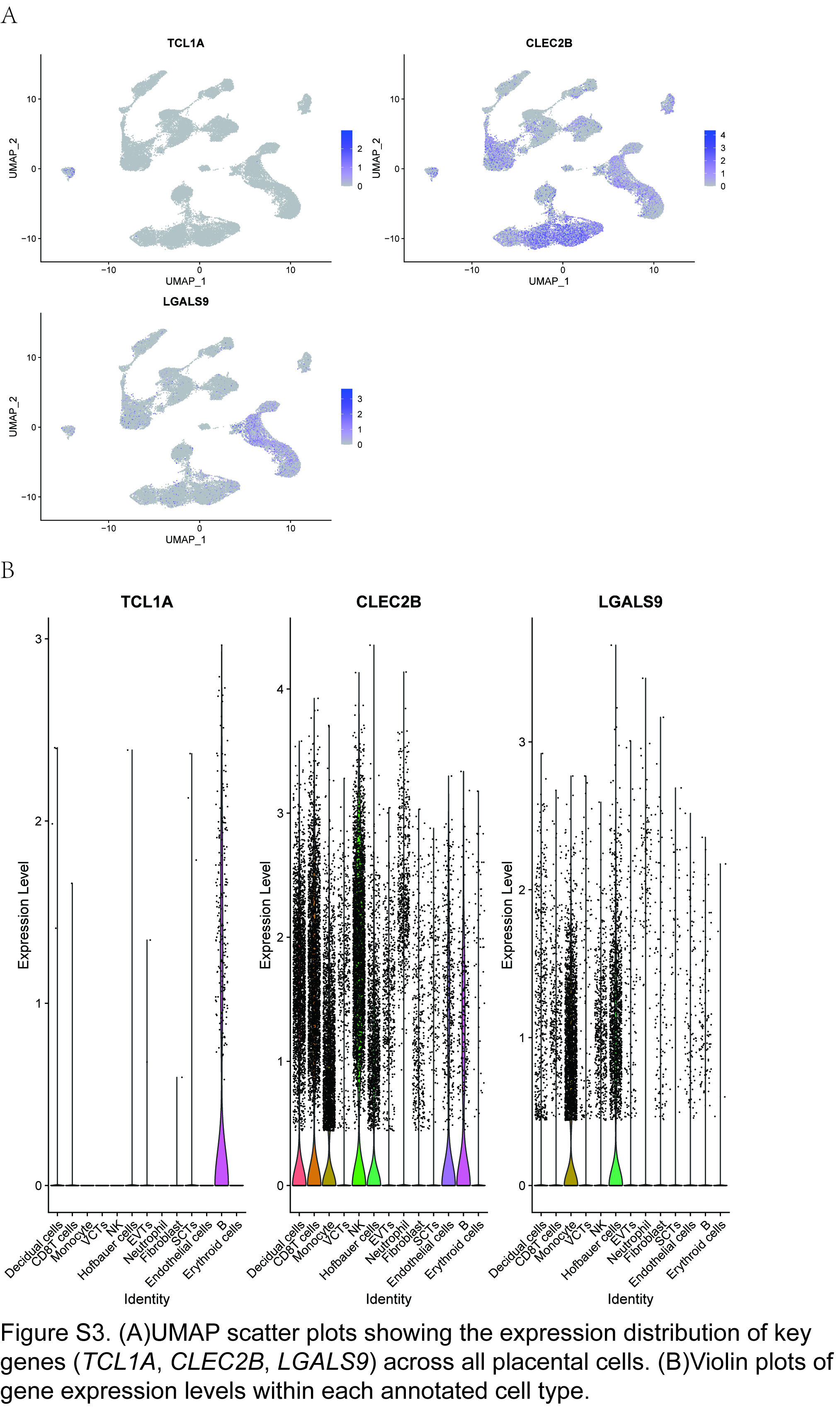

Supplement: Supplementary file 3 [file Image3.tif]
